# Supplementary material for: Serum high sensitivity C-reactive protein poorly predicts bone mineral density: A NHANES 2017–2020 analysis
Source: PLoS One. 2023 Oct 12;18(10):e0288212. doi: 10.1371/journal.pone.0288212 (PMC10569631; doi:10.1371/journal.pone.0288212)
Supplement: Attachment — Submitted filename: Response to Reviewers.docx [file pone.0288212.s002.docx]

**Rebuttal Letter: PONE-D-23-14590**

*Serum high sensitivity C-reactive protein poorly predicts bone mineral density: a NHANES 2017-2020 analysis*

I thank the reviewer for their thoughtful review and suggested revisions. Please find below a point-by-point response. I hope you find all comments to be adequately addressed.

**Reviewer Comment #1:** please add keywords.

- The following keywords have been added to the manuscript on Page 1, Line 19: osteoporosis, fracture risk, diverse population

**Reviewer Comment #2:** it would be appropriate to briefly define the objectives of the analysis in a separate paragraph.

- The following sentence defining the objectives of the analysis have been added on Page 4, Lines 80-82:

“The specific objectives of this analysis were to determine if, and how, the sexual and racial diversity of the sample population impacts the correlative and predictive relationships between CRP and bone mineral density and fracture risk.”

**Reviewer Comment #3:** summarizing the results of the discussion in the form of conclusions in a separate paragraph.

- The following paragraph summarizing the results of the discussion in the form of conclusions has been added to Pages 14-15, Lines 322-332.

“In conclusion, serum levels of C-reactive protein statistically correlate with and predict femoral neck and spine BMD in a sexually and racially diverse population. However, as the magnitude is too low to be biologically meaningful, significant caution should be taken in interpreting the clinical significance and application of serum C-reactive protein as a biomarker for osteoporosis. Weight more robustly predicts femoral neck and spine BMD, compared to serum C-reactive protein. Given that overweight and obese individuals account for nearly half of all osteoporotic fractures, the predictive power of weight in identifying individuals at risk for osteoporosis is severely limited. Current research should continue efforts to identify a clinically available biomarker that robustly predicts low bone mineral density and fracture risk in a diverse population and across of range of body weights and compositions. Rates of both osteoporosis and obesity continue to increase, with osteoporosis remaining largely undetected prior to first fracture, lending significant credence to the need for a robust and predictive biomarker.”
